# Surgical management for a huge presacral teratoma and a meningocele in an adult with Currarino triad: a case report

**DOI:** 10.1186/s40792-018-0419-2

**Published:** 2018-01-19

**Authors:** Shigenobu Emoto, Manabu Kaneko, Koji Murono, Kazuhito Sasaki, Kensuke Otani, Takeshi Nishikawa, Toshiaki Tanaka, Keisuke Hata, Kazushige Kawai, Hideaki Imai, Nobuhito Saito, Hiroshi Kobayashi, Sakae Tanaka, Masako Ikemura, Tetsuo Ushiku, Hiroaki Nozawa

**Affiliations:** 1 Department of Surgical Oncology, The University of Tokyo, 7-3-1, Hongo, Bunkyo-ku, Tokyo, 113-8655 Japan; 2 Department of Neurosurgery, The University of Tokyo, Tokyo, Japan; 3 Department of Orthopaedic Surgery and Spinal Surgery, The University of Tokyo, Tokyo, Japan; 4 Department of Pathology, The University of Tokyo, Tokyo, Japan

**Keywords:** Currarino triad, Presacral teratoma, Meningocele

## Abstract

**Background:**

The Currarino triad is a rare hereditary syndrome comprising anorectal malformation, sacral bony defect, and presacral mass. Most of the patients are diagnosed during infancy.

**Case presentation:**

A 44-year-old man was diagnosed with Currarino triad, with a huge presacral teratoma and meningocele. One-stage surgery via posterior approach was successful.

**Conclusions:**

Treatment of the presacral mass in the Currarino triad, diagnosed in adulthood, is challenging. Multidisciplinary management and detailed planning before surgery are important for a satisfactory outcome.

## Background

The Currarino triad was first described by Currarino et al. in 1981 [1]. This syndrome is an autosomal dominant condition resulting from mutations in the homeobox HLXB9 gene on chromosome 7. It comprises an anorectal malformation, a sacral bony defect, and a presacral mass. This congenial syndrome is thought to be caused by malformation of the caudal notochord, which leads to aberrant secondary neurulation with incomplete separation of the ectodermal and endodermal layers in the developing embryo [2]. In more than 80% of cases, this triad is diagnosed in infancy. Herein, we present a rare case of the Currarino triad, which was diagnosed in mid-forties with constipation secondary to the huge presacral teratoma, with meningocele, and was successfully treated with one-stage surgery.

## Case presentation

A 44-year-old man complaining of intermittent constipation for 3 years presented to his local hospital with an increased severity of constipation of 3-month duration. Computed tomography (CT) scan showed a large presacral mass measuring 9 cm in diameter, and he was then referred to our hospital. His past medical history was significant for colostomy performed just after birth for anal atresia and anorectoplasty with stoma closure when he was 3 years old. He had occasionally, several times a year, suffered from incontinence. In his family history, his father died of lung cancer at the age of 65, and his mother was 80 years old and healthy. He had no siblings or children. He had no family history of hereditary diseases. He was not receiving regular medications.

Physical examination of the abdomen showed a surgical scar extending from the left upper to lower quadrants without palpable masses. At the perineum, the rectal mucosa was anastomosed to the skin by the previously performed anoplasty. Digital rectal examination showed that the aperture of the rectum (plastic anus) was hard and that the lumen of the rectum was narrow. An elastic hard presacral mass was palpable at 4 cm from the anal verge. He could voluntarily constrict the anal sphincter. The rest of the physical examination, including urinary and genital systems, did not reveal any abnormalities.

Laboratory analyses were normal, including tumor markers (carcinoembryonic antigen, carbohydrate antigen 19–9, anti-p53 antibody, alpha-fetoprotein, prostate-specific antigen, neuron-specific enolase, and squamous cell carcinoma antigen).

Anorectal manometry revealed an impaired anal function; the maximum static pressure was 22 mmHg (normal range 26–64 mmHg), and the voluntary contraction pressure was 43 mmHg (normal range 59–206 mmHg).

Colonoscopy and contrast enema showed that the lower rectum was stenotic but with intact mucosa.

**Fig. 1:**
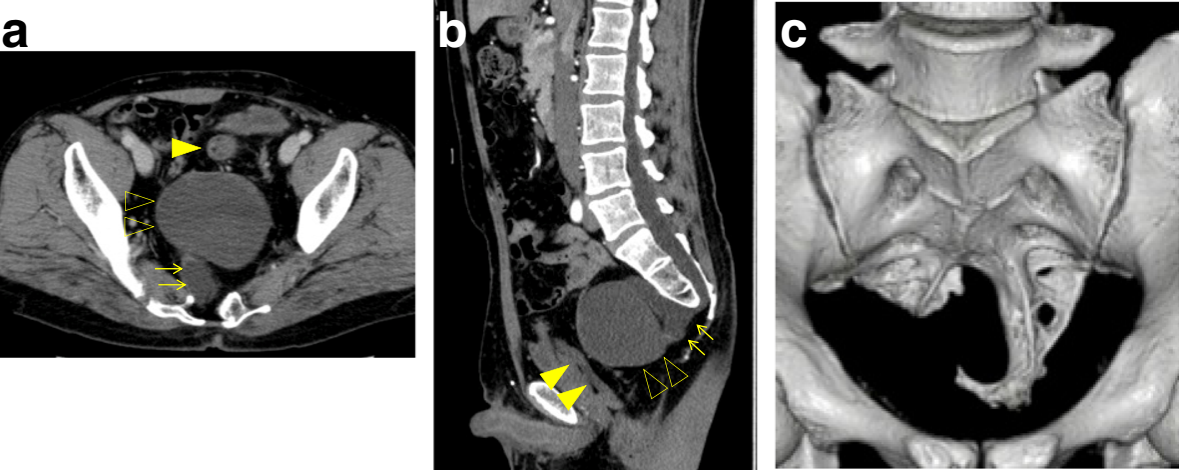
Computed tomography (CT) scan showing a 60 × 30 × 21-mm meningocele (→) which was protuberated anteriorly from the level of S3; a 90 × 85 × 68-mm mass (△) which was located anterior to the meningocele; and a dislocated rectum (▲) (**a**, **b**). A partial defect of the sacrum with deformity was detected (**c**)

**Fig. 2:**
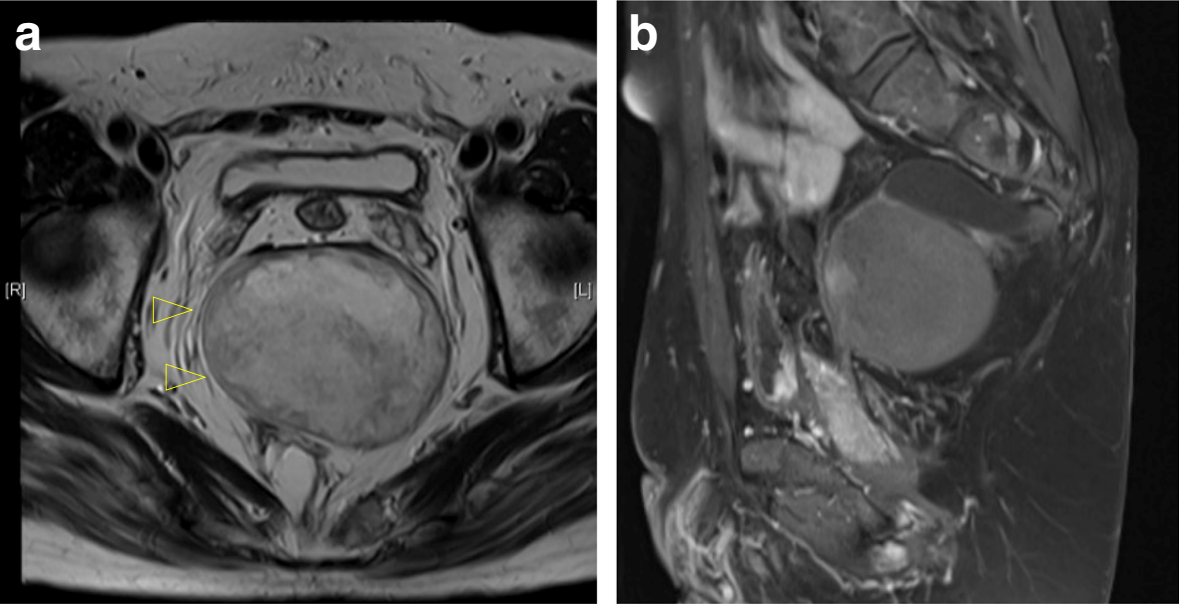
Magnetic resonance imaging (MRI). The inside of the cystic tumor (△) showed heterogeneously high intensity in T2-weighted image (**a**) and homogenously low intensity in T1-weighted image. The wall was enhanced by Gadolinium (**b**)

CT scan showed a partial defect in the right side of the sacrum with deformity, a 60 × 30 × 21-mm meningocele which was protuberated anteriorly from the level of the S3 vertebra, and a 90 × 85 × 68-mm cystic mass which was located anterior to the meningocele, compressing the rectum ventrally (Fig. 1). In addition, double renal pelvises were observed in the right kidney, and the inferior mesenteric artery branched from the superior mesenteric artery. On magnetic resonance imaging (MRI), the inside of the cystic tumor showed heterogenous high intensity in T2-weighted image, and an almost homogenous low intensity in T1-weighted image, with the wall showing enhancement with Gadolinium (Fig. 2). The existence of the fat component highly suggested teratoma as a preoperative diagnosis. The levator ani muscle appeared intact, except for partial atrophy.

Fluorodeoxyglucose-positron emission tomography showed no abnormal uptake. Myelography showed an inflow of the contrast agent into the meningocele, but not into the presacral tumor.

**Fig. 3:**
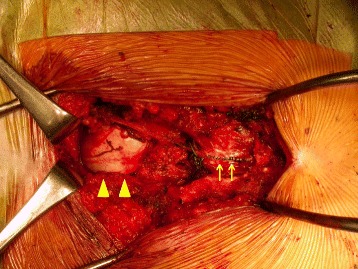
S3-4 laminectomy was performed, the dura was opened, and the orifice of the meningocele was sutured and dissected. After the dura was closed (arrow), we exposed the capsule of the teratoma (▲)

Resection of the presacral mass was performed, based on a diagnosis of Currarino syndrome associated with presacral teratoma and meningocele. A postsacral midline incision was made with the patient in the jackknife position. After S3-4 laminectomy, the meningocele was dissected by ligating the root at the level of the dura. Then, the removal of the coccyx with an extended incision provided excellent visualization of the teratoma (Fig. 3). The posterior wall of the most distal area of the rectum was severely adherent, resulting in tumor rupture during dissection. We completely removed the clay-like content and the capsule. The peritoneal cavity was not opened. The wave of the anal sphincters, monitored by motor-evoked potential (MEP), did not attenuate throughout the operation. The operation time was 8 h 56 min and intraoperative blood loss was 430 ml (including spinal fluid).

**Fig. 4:**
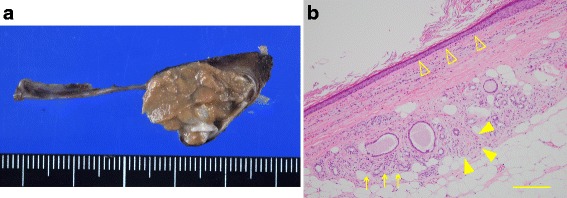
Macroscopic appearance (**a**) and microscopic findings (**b**) of a section of the resected teratoma. The cyst wall was mainly composed of keratinizing stratified squamous epithelium (△). Fat, sweat glands (arrows), and peripheral nerves (▲) were observed around the cyst wall, and the pathological diagnosis was mature teratoma. The scale bar represents 200 μm

Pathological examination revealed a mature teratoma (Fig. 4). The cyst wall was composed of keratinizing stratified squamous epithelium surrounded by fat, sweat glands, and peripheral nerves. The content, 200 g in weight, was composed of a histologically keratinized substance. The coccyx showed no abnormality.

Oral intake was started on the fourth postoperative day. He was discharged from the hospital on the 15th postoperative day with no morbidity. His constipation dramatically improved postoperatively. No urological or rectal complications, or lower extremity neuropathy, were observed.

## Discussion

The presacral mass associated with the Currarino triad is reportedly a teratoma, hamartoma, neuronteric cyst, anterior meningocele, or a combination of these lesions [3]. Treatment of the anorectal anomaly and the presacral tumor can be performed by one-step posterior sagittal anorectoplasty. If a meningocele exists, it is usually removed independently from the procedure for the anorectal malformation and presacral mass [4]. For early diagnosis of Currarino syndrome, if an anorectal anomaly is identified in a child, the existence of the other two components of the triad should be considered. In our case, only atresia ani was found and treated during childhood, while the presacral mass was not recognized until adulthood, when it gradually grew causing severe constipation.

Posterior, transabdominal, and combined approaches were previously reported for the surgical resection of presacral tumors. Table 1 summarizes the features of these approaches. Posterior approach can be applied when the tumor diameter is less than 10 cm [5, 6]. The advantages of the posterior approach include easy accessibility, direct dissection around the coccyx or levator ani muscle, and avoidance of peritoneal dissemination of the tumor cells and adhesive intestinal obstruction. However, there is a risk of injury to the hypogastric nerves, and median sacral vessels, if the tumor extends into the upper pelvis, with subsequent difficulty in exposing the cranial side of the tumor.

**Table 1:** Advantages and disadvantages of each approach for presacral tumor resection

	Posterior approach	Transabdominal approach
Advantages	- Short distance to the tumor. - Direct dissection around the coccyx or levator ani muscle. - Coccygectomy can be added.	- Cranial side of large tumors (> 10 cm) can be exposed easily.
Disadvantages	- Risk of injury to hypogastric nerves and median sacral vessels. - Exposure of the cranial side of the tumor is difficult.	- Dissection around the lower rectum is difficult. - Risk of injury to proximal part of the mesentery (which may require resection of the rectum and/or stoma creation). - Risk of peritoneal dissemination. - Risk of adhesive intestinal obstruction.

In our case, a posterior approach was the best to avoid spinal leakage or possible meningitis caused by injuring the meningocele. Closure of the meningocele from the posterior side was necessary before removal of the teratoma. In addition, adhesion between the teratoma and the lower rectum was expected because surgery for anal atresia was performed during childhood. The partial defect of the sacrum, as in our case, also favors the posterior approach. Though there are some reports of laparoscopic en bloc resection of teratoma [7, 8], rupture of the wall or aspiration of the contents during resection was also reported [9, 10]. Rupture of the free peritoneal cavity should be avoided as occasionally the presacral teratoma in Currarino syndrome is malignant [11, 12].

Adding coccygectomy to the resection of congenital presacral tumors remains controversial. Traditional treatment of sacrococcygeal teratomas favors removing the coccyx because the totipotential cells are believed to arise from the coccyx [13]. However, complications associated with coccygectomy include delayed wound healing, increased wound infection rate, prolonged recovery time, and rarely, bowel herniation. Thus, in recent case reports, coccygectomy was sometimes omitted when the presacral tumor was located away from the coccyx [12, 14, 15]. In our case, resection of the coccyx was performed in order to expose and resect the tumor via the posterior approach.

## Conclusions

In conclusion, although the treatment of the presacral mass with Currarino triad diagnosed in adulthood is challenging, multidisciplinary management can allow feasible removal of the tumor via the posterior approach.

## Abbreviations

CT: Computed tomography
MEP: Motor-evoked potential
MRI: Magnetic resonance imaging
